# Isolation and high-dimensional flow cytometric analysis of tumor-infiltrating leukocytes in a mouse model of colorectal cancer

**DOI:** 10.3389/fimmu.2024.1295863

**Published:** 2024-03-04

**Authors:** Christina Eich, Johannes F. Vogt, Vivian Längst, Björn E. Clausen, Nadine Hövelmeyer

**Affiliations:** ^1^ Institute for Molecular Medicine, Paul Klein Center for Immune Intervention, University Medical Center of the Johannes Gutenberg-University Mainz, Mainz, Germany; ^2^ Research Center for Immunotherapy (FZI), University Medical Center of the Johannes Gutenberg-University Mainz, Mainz, Germany

**Keywords:** azoxymethane/dextran sodium sulfate-induced cancer mouse model, colitis-associated cancer, dendritic cells, flow cytometry, macrophages, myeloid cells, tumor immunology, tumor microenvironment

## Abstract

Colorectal cancer (CRC) is a complex and heterogeneous disease characterized by dysregulated interactions between tumor cells and the immune system. The tumor microenvironment plays a pivotal role in cancer initiation as well as progression, with myeloid immune cells such as dendritic cell and macrophage subsets playing diverse roles in cancer immunity. On one hand, they exert anti-tumor effects, but they can also contribute to tumor growth. The AOM/DSS colitis-associated cancer mouse model has emerged as a valuable tool to investigate inflammation-driven CRC. To understand the role of different leukocyte populations in tumor development, the preparation of single cell suspensions from tumors has become standard procedure for many types of cancer in recent years. However, in the case of AOM/DSS-induced colorectal tumors, this is still challenging and rarely described. For one, to be able to properly distinguish tumor-associated immune cells, separate processing of cancerous and surrounding colon tissue is essential. In addition, cell yield, due to the low tumor mass, viability, as well as preservation of cell surface epitopes are important for successful flow cytometric profiling of tumor-infiltrating leukocytes. Here we present a fast, simple, and economical step-by-step protocol for isolating colorectal tumor-associated leukocytes from AOM/DSS-treated mice. Furthermore, we demonstrate the feasibility of this protocol for high-dimensional flow cytometric identification of the different tumor-infiltrating leukocyte populations, with a specific focus on myeloid cell subsets.

## Introduction

### Colorectal cancer

Colorectal cancer (CRC) is the third most prevalent cancer worldwide, accounting for approximately 10% of all cancer cases. Current standard-of-care therapies like chemotherapy, radiotherapy, and surgical resection have been mainstays in managing the disease but improve survival in only up to 20% of patients. Despite advances in the detection and treatment of CRC in recent years, CRC has remained a major challenge in clinical practice and there is a growing interest in understanding the role of the immune system in combating CRC. This malignant tumor of the intestinal tract can arise spontaneously (sporadic CRC) or as a result of chronic inflammation, known as colitis-associated colorectal cancer (CAC). Patients with inflammatory bowel diseases (IBD) such as Ulcerative colitis or Crohn´s disease are at a significantly higher risk to develop CRC (1).

The tumor tissue represents an intricate system consisting not only of malignant cells, but also of surrounding stroma and, in particular, a complex tumor immune cell infiltrate. This includes cells of the innate immune system like dendritic cells (DC), macrophages (Mph), neutrophils, and myeloid-derived suppressor cells (MDSC), as well as adaptive immune cells (T and B cells) (1, 2). These diverse cell types contribute to the inflammatory status of the tumor tissue and communicate with each other directly via cell-cell contact or indirectly through cytokine and chemokine production to shape tumor growth. In particular, improved prognosis of CRC and shorter patient overall survival are associated with high and low levels of anti-tumor T cell activity, respectively. Interestingly, while the activity of tumor-specific T cells decreases as the tumor progresses, the density of innate immune cells and of B cells increases, suggesting potent immunosuppressive mechanisms in the tumor microenvironment (TME) (3, 4). Therefore, understanding the multifaceted interactions between CRC and the immune system holds great potential for innovative therapies and improved outcomes.

### Intestinal immune system: a double-edged sword in CRC development

The intestinal immune system plays an important role in the development and progression of CRC (1). Antigen presenting cells (APC) constitute a heterogeneous population of cells acting as sentinels of the immune system. The main types of APC are DC, Mph, and B cells. DC are broadly classified as plasmacytoid DC (pDC) and conventional DC (cDC), each with specialized functions. pDC are characterized by their ability to produce large amounts of type I interferon (IFN-I) upon viral infection (5), yet their role in anti-tumor immunity remains to be fully explored. However, intratumoral pDC appear to exhibit impaired IFN-I production and immunosuppressive properties. cDC are the most potent type of APC and their ability to capture, process, and present antigens to naïve T cells makes them unique initiators and regulators of tumor-specific adaptive immune responses (6). Mph on the other hand are pivotal to maintain mucosal tissue homeostasis *in situ*, but tumor-associated Mph (TAM) can also promote chronic inflammation and tumor growth (7, 8). Beyond secreting IgA to maintain homeostasis with the microbiota in the gut lumen, and tumor-specific IgG1 antibodies, B cells present tumor antigens via MHCI or MHCII to T cells, enhancing their anti-tumor effector function (9). Notably, all of these three APC populations represent double-edged swords when it comes to CRC development, because the TME constitutes a special immunosuppressive milieu facilitating tumor immune evasion.

### Myeloid APC: orchestrators of anti-tumor immunity

Myeloid APC populations that shape tissue homeostasis and orchestrate adaptive immune responses via the secretion of soluble mediators comprise Mph and cDC. Mph primarily maintain tissue homeostasis *in situ* and represent a crucial immune cell population and key regulators within the TME. They demonstrate a remarkable plasticity and can display diverse phenotypes depending on the microenvironmental cues. TAM are the most common myeloid cell type in the TME and are recruited to the tumor tissue by chemokines released from cancer cells. So called M1-like Mph, identified by expression of Nos2, exhibit an anti-tumor phenotype and secrete pro-inflammatory cytokines, such as IL-12 and TNF-α. These cytokines promote T cell activation and anti-tumor immunity, making M1-like Mph critical for the early stages of the immune response against CRC. On the other hand, M2-like Mph characterized by Arg1 expression display an immunosuppressive phenotype and release anti-inflammatory cytokines such as IL-10 and TGF-β (10). They also contribute to tissue remodeling, angiogenesis, and the resolution of inflammation. Thus, in the context of cancer, M2-like Mph can promote tumor growth and progression by creating an immunosuppressive microenvironment and facilitating tumor invasion and metastasis. In contrast to other solid tumors, TAM infiltration in CRC failed to predict outcome (11) or correlate with a better prognosis (12). Notably, clear division into M1- and M2-like Mph subsets by the use of genes such as *Nos2* or *Arg1* often fails in the context of cancer, especially colorectal cancer (13, 14). Therefore, it is of great interest to identify new marker genes which allow classification of TAM into pro-tumorigenic and anti-tumorigenic.

cDC, on the other hand, are unique initiators and regulators of adaptive immune responses, including potent anti-tumor immunity as well as tumor immune evasion (15, 16). In this context, CD8^+^/CD103^+^ cDC1 excel at inducing cellular immunity against intracellular pathogens and tumors due to their efficient cross-presentation of exogenous antigens on MHCI molecules to activate naïve CD8^+^ T cells and their ability to prime T helper (Th) 1 cell responses. cDC1 have evolved unique biological properties, including using the lectin Clec9A/DNRG1 to take up dead cells, and transport cell-associated material into endocytic compartments specialized for cross-presentation (17). Instead, CD11b^+^ cDC2 are crucial for inducing CD4^+^ T cell-mediated immunity, i.e., in cancer (18). In addition, CD4^+^ T cells can engage with cDC1 via CD40L/CD40 signaling ‘licensing’ them to cross-prime CD8^+^ T cells (19, 20). cDC, however, do not only initiate and polarize immune responses to a given (pathogenic) threat, but they are also responsible for maintaining immune (self-) tolerance. A shift in this balance towards excessive immune reactivity or an unwarranted tolerogenic function can lead to chronic inflammation (and autoimmunity) or facilitate unchecked tumor development.

Indeed, secretion of soluble tumor-derived factors that create a suppressive TME resulting in cDC and Mph dysfunction have long been described as key mechanisms of cancer immune evasion (21, 22). Specifically, the TME contains a network of regulatory factors that can inhibit cDC infiltration and subdue their anti-tumor activity. The former includes reduced CCL4, CCL5, and XCL1 chemokine as well as Flt3L expression limiting cDC recruitment and differentiation, respectively. The latter involves IL-6 and IL-10 overexpression, which enforces immune-regulatory transcriptional programs and limits cDC differentiation and maturation. Although the original hypothesis stated that Mph are involved in anti-tumor immunity, there is substantial evidence that TAM can enhance tumor progression (23). Mph chemotaxis is regulated by CCL2, which is overexpressed in CRC. Despite extensive research, the exact cellular and molecular mechanisms underlying these complex processes orchestrating Mph and cDC function during tumor immune surveillance and escape remain elusive.

### Tumor-infiltrating lymphocytes: crucial effector cells controlling tumor growth

As outlined above, naïve T cells are instructed by cDC in tumor-draining lymph nodes and activated effector T cells that subsequently infiltrate the tumor are reactivated by resident APC, in particular Mph *in situ* (24). Notably, adaptive immune responses to CRC are modulated by the TME, including TME-conditioned migratory cDC, and the locations and interactions of immune cells in the colorectal TME leading to dysregulation of these cell populations are complex and heterogeneous (25). In general, Th1 and cytotoxic T cell responses correlate with better outcomes of patients, whereas Th17 and regulatory T (Treg) cell responses have been associated with worse prognosis (26). Protection is mediated by the anti-proliferative, pro-apoptotic, and anti-angiogenic actions of IFN-γ, as well as through enhanced recruitment of cytotoxic CD8^+^ T cells. On the other hand, IL-17A stimulates tumor development and progression directly as well as indirectly by inducing secretion of IL-6 by APC (27). IL-17A also promotes angiogenesis via production of vascular endothelial growth factor (VEGF) (28). In contrast, IL-17F has a tumor suppressive effect in CRC, possibly by inhibiting tumor angiogenesis, and *Il17a*- and *Il17f*-deficient mice develop fewer and more tumors, respectively, compared to littermate controls in the AOM/DSS model (29). Finally, Treg cells, in particular CD4^+^CD25^+^Foxp3^+^ Treg cells, play critical roles in establishing and maintaining an immunosuppressive TME to inhibit anti-tumor immunity. On one hand, these Treg cells express inhibitory receptors such as CTLA-4, Tim-3, and PD-1 that exert their suppressive function on both cDC and Mph. In addition, Treg cells secrete the immunosuppressive cytokines IL-10 and TGF-β to induce APC and effector T cell dysfunction (30). However, IL-10 represents a pleiotropic cytokine and whether it is a tumor-promoting or -inhibiting agent is context dependent and still requires further investigation.

B cells are also an important part of the tumor immune cell infiltrate in CRC, and their contribution to tumor initiation, development, and immune surveillance is complex with both pro- and anti-tumorigenic effects. Recent studies implicate a fundamental role of B cells in shaping anti-tumor responses through several mechanisms. While IgA^+^ plasma cells in general regulate bacterial populations in the gut lumen, for example by providing a protective barrier between commensals and the epithelium, plasma cell-derived tumor-specific IgG1 antibodies mediate cell cytotoxicity, and phagocytosis of tumor cells. On the other hand, IgA^+^ plasma cells turned out to be a source of IL-10 and PD-L1, causing suppression of anti-tumor Th1 cells and CTL (31). In addition, B cells present tumor-specific antigens via MHCII to Th cells inducing their anti-tumor effector function and they also regulate the immune response within the TME through the release of cytokines, such as IFN-γ, IL-12, or IL-10 (32, 33). B cells constitute a significant proportion of the immune cell infiltrate in CRC where CCR6^+^ B cells are actively recruited to the TME (34). CRC patients show substantial alterations in their B cell compartment, with increasing numbers of IL-10 producing B cells in advanced tumors and metastasis (35). Otherwise, CRC patients with tumors heavily infiltrated by CD20^+^ B cells showed significantly improved disease-specific survival, suggesting an anti-tumor role for B cells. These B cells are strongly associated with CD8^+^ cytotoxic T cells, which are pivotal in antigen-specific immunity against tumors (4).

### AOM/DSS model of inflammation-associated cancer

Although major improvements in CRC screening and treatment have been made in recent years, improved strategies to combat CRC remain an important clinical need. The Azoxymethane (AOM)/Dextran sodium sulfate (DSS) model is a powerful, reproducible tool to better understand the mechanisms underlying genesis and progression of CAC (36). The combination of AOM (tumor-inducing agent) with the inflammatory agent DSS (tumor-promoting agent) triggers CAC tumor development within 10 weeks. AOM is a procarcinogen that is metabolized in a cytochrome P450-dependent manner in the liver, which results in its activation. Active metabolites are released into the intestine by excretion via the bile. In the gut, contributions from the intestinal flora promote further activation of AOM derivatives to methyldiazonium, which in turn mediates colonotropic mutagenicity (36). DSS, a heparin-like polysaccharide that inflicts damage to the colonic epithelium, triggers colitis that mimics some of the features of IBD, including bloody diarrhea, intestinal inflammation, weight loss, and shortening of the colon, and is thought to promote tumor formation. Tumors induced in mice exposed to AOM/DSS accurately recapitulate the pathogenesis observed in human CAC. They often begin with polypoid growth and occur very frequently in the distal part of the colon, which is also the predominant site in patients (37), although AOM/DSS-induced tumors lack mucosal invasiveness and have a very low tendency to metastasize (38). Accordingly, the mice develop only adenomas, representing early disease, whereas the carcinomas in human CAC are late disease. Thus, the AOM/DSS-model allows for the analysis of tumors to study the impact of the TME on subversion of anti-tumor immunity.

### Objective and purpose of this protocol

Preparation of single cell suspensions from AOM/DSS-induced colorectal tumors is challenging and has rarely been described. Here, we provide a simple protocol for the isolation of CD45^+^ leukocytes, especially myeloid cells, from colorectal tumors induced by AOM/DSS treatment in mice. This protocol not only addresses the issues of cell viability and preservation of cell surface epitopes, but also emphasizes fast cell extraction. We have further validated the feasibility of this protocol for high-dimensional flow cytometric analysis, with a particular focus on comprehensive identification of myeloid cell subsets. With this protocol, we aim to provide researchers and clinicians with a robust and easy to follow method to dissect the intricate immune landscape of AOM/DSS-induced colorectal tumors. A deeper understanding of the role of different leukocyte populations, in particular myeloid APC, in CAC forms the basis for the development of novel therapeutic strategies for this complex and heterogeneous disease.

## Material and equipment

## Methods

### Induction of inflammation-associated colorectal tumor growth in mice

**Figure 1:**
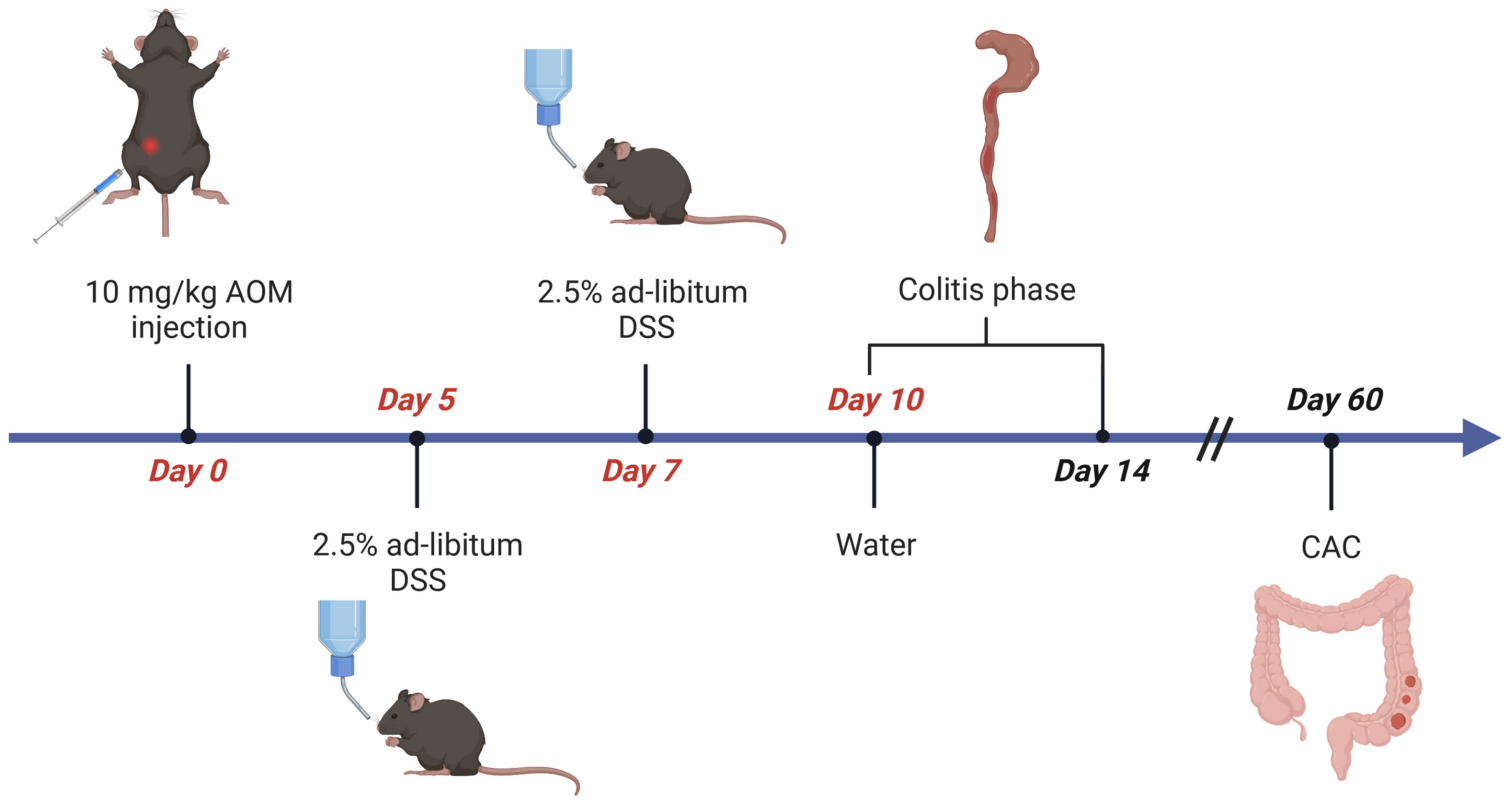
Flow chart of AOM/DSS-induced CRC in mice. Age- and sex-matched littermates are injected intraperitoneally (i.p.) with AOM (10µg/g body weight) on day 0. From day 5 to day 10 mice receive 2,5% (w/v) dextran sulfate sodium (DSS) solved in autoclaved drinking water ad libitum, which is refreshed on day 7. Afterwards, the animals receive normal water for the rest of the experiment. Mice are sacrificed on day 14 for evaluation of inflammation, or on day 60 for analysis of CAC. Figure created with Biorender.com.

To induce CAC, on day 0, cohorts of 6-8 week-old, sex-matched wild type C57BL/6 mice are injected intraperitoneal (i.p.) with the procarcinogen Azoxymethane (AOM in PBS, 10mg AOM per kg body weight) (**Table 1**). From day 5 to 10, mice receive one cycle of 2.5% Dextran Sodium Sulfate (DSS, 40-50kDa) in autoclaved drinking water (**Tables 1**, **2**). As DSS degrades over time, it is recommended to replace the DSS solution on day 7. The addition of DSS facilitates tumor initiation to some extent and further promotes tumor growth by driving intestinal inflammation (**Figure 1**), resulting in transient weight loss. Acute, chronic, and relapsing models of intestinal inflammation can be achieved by modifying the concentration as well as the frequency of DSS administration (37). From day 10, the completion of the DSS cycle, until the end of the experiment, the mice are provided with regular autoclaved drinking water. Each mouse needs to be monitored for body weight, general condition, clinical abnormalities, and any sign of discomfort in accordance with the specific ethical regulations at the investigator´s site. Most animals display a temporary weight loss of 10% at the peak of a DSS cycle and should be fully recovered within 3-4 days. Weight loss greater than or equal to 20% of initial body weight is a termination criterion and the animal should be euthanized (according to institutional guidelines). Next to weight loss, mice frequently display soft stool or even bloody diarrhea during DSS treatment. Clinical signs of inflammation can be assessed endoscopically on day 14 using an appropriate grading system such as the MEICS score (39). Mice are euthanized on day 60 of the AOM/DSS protocol for tumor analysis, when the animals no longer show signs of inflammation. Due to the inherent high variation in tumor burden, we recommend including at least 8 mice per test group in an experiment.

**Table 1:** List of used reagents.

Reagent	Manufacturer	Catalogue number
Fetal Calf Serum (FCS)	Sigma	F7524
Phosphate Buffered Saline (PBS) without calcium and magnesium	Sigma	D8537
Ethylendiamintetraacetat (EDTA) (0.5M)	Sigma	E5134-500G
Collagenase IV	Worthington	LS0004186
Deoxyribonuclease I (DNaseI)	Roche	10104159001
Rotihistofix (4% formaldehyde (FA), pH7)	Carl Roth GmbH + Co.KG	P087.2
Trypan blue	Gibco	15250-061
Azoxymethane (AOM)	Sigma-Aldrich	A5486
Dextran sulfate sodium (DSS) salt, colitis grade	MPbio	160110

**Table 2:** Buffer composition.

Name of buffer	Ingredients	Final concentration
Digestion mix	RPMI (with glutamine)	
	Collagenase IV	200U/mL
	0.5 U/mL DNase I	0.5U/mL
PBS/EDTA solution	PBS	
	EDTA	2mM
FACS buffer	PBS	
	FCS	3%
	EDTA	2mM
DSS solution	Autoclaved water	
	DSS	2.5%

### Isolation of AOM/DSS-induced tumor tissue

**Figure 2:**
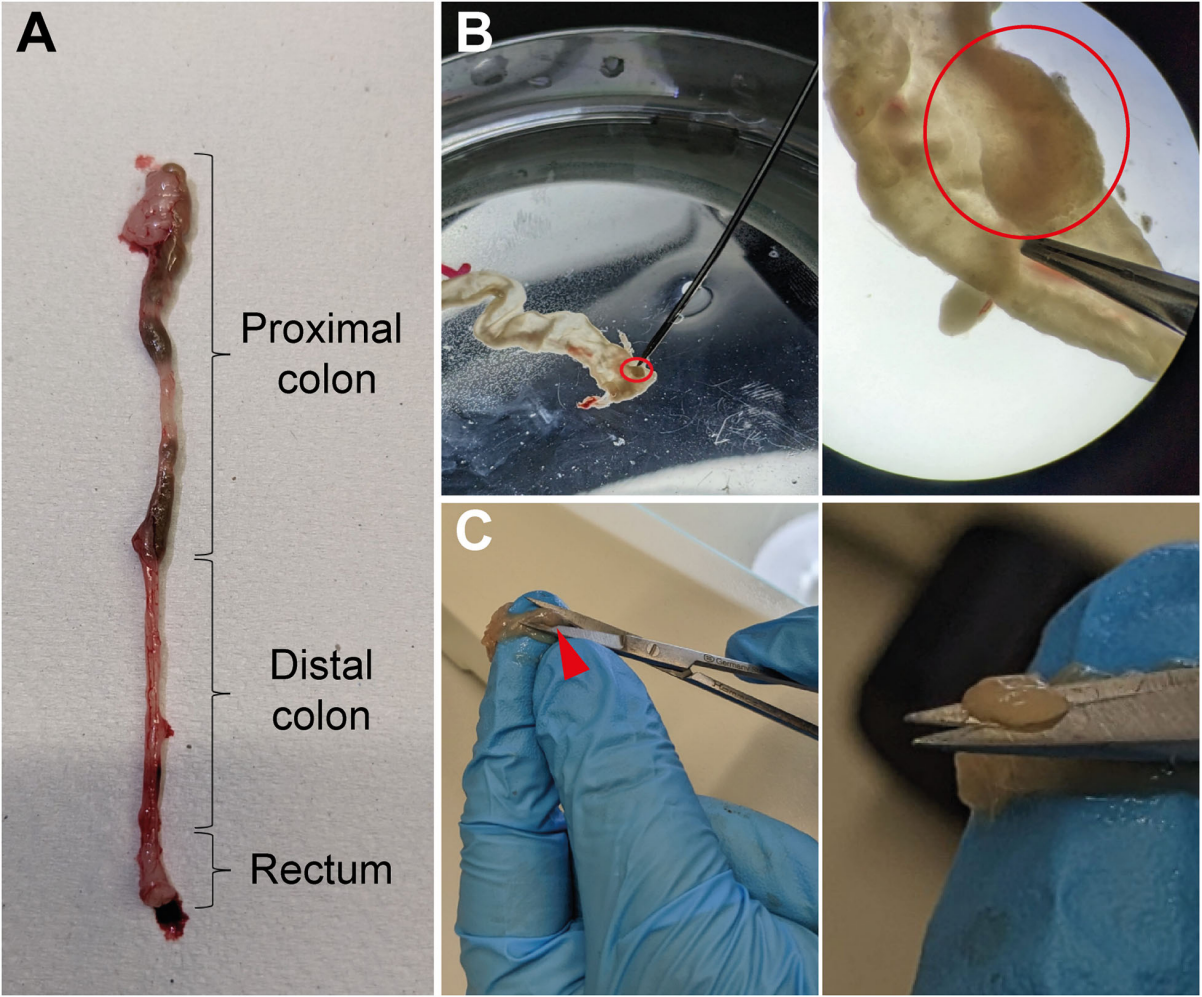
Isolation of tumors from colon tissue. **(A)** Image of a murine colon with the different parts marked. **(B)** Longitudinally opened colon tissue in a PBS-filled petri dish with a tumor highlighted (red circle, upper middle panel) and the same biopsy as it appears through a microscope with the same tumor circled in red (upper right panel). **(C)** Illustration of the procedure in which the colon tissue is pushed through the fingers of one hand and the tumors (red arrowhead, lower middle panel) are excised with fine scissors (higher magnification, lower right panel).

Set up fine forceps and scissors, two petri dishes filled with cold PBS, 1.5mL tubes, laminated graph paper, camera (**Tables 1**, **3**). To collect the tumors, mice are euthanized on day 60 of the AOM/DSS protocol, and the colon is isolated from the abdominal cavity (**Figure 2A**). Fat must be removed by holding the colon in place with one hand and very gently pulling the fat off with a fine forceps. Afterwards, the colon is opened longitudinally with a scissor, ideally with blunted tips to prevent ripping of the intestinal wall. Each colon is washed in a petri dish filled with cold PBS by quickly but gently moving the colon back and forth through the PBS with tweezers to remove fecal matter. Subsequently, the opened colon is transferred into a fresh PBS-filled petri dish to examine it for tumors under a microscope (**Figure 2B**, *upper panel*). The colon tissue should be carefully stretched using fine forceps and thoroughly scored for tumors from the distal to the proximal end. Tumors appear as round, dense structures (**Figure 2B**, *lower panel*). After the tumors have been identified, the colon tissue is hold in place with one hand and each tumor is meticulously excised with fine scissors (**Figure 2C**), transferred into a 1.5mL tube filled with 100µL cold PBS, and immediately placed on ice. Repeat until all tumors from one mouse are collected in one tube (in our hands, tumor incidence for C57BL/6 mice is 3-12 tumors per mouse). For tumor area measurement, place the tumors on laminated graph paper and take pictures, preferably at high resolution (for calculation of tumor area, see results section ‘Quantification of tumor burden and size’).

**Table 3:** Overview of equipment and consumables.

Equipment	Manufacturer	Catalogue number
1.5mL tubes	Sarstedt AG	72.690.001
50mL tubes	Greiner bio-one	227261
70µm cell strainer	Falcon	10082019
96-well plate (V shape)	Thermo Scientific	163320
BD FACSSymphony™	BD Biosciences	
Centrifuge “Z 446 K”	Hermle Labor Technik	6.268 644
Forceps & Scissors	HSB Hammbacher	HSB-390-10 (51807020)
Neubauer chamber	Superior Marienfeld	0640010
Petri dishes	Corning™	15458784
Pipetboy	Fisher Scientific	11701258
Stereomicroscope Leica M80	Leica	
Thermo shaker MKR13	DITABIS AG	HA02.1

### Single-cell suspension of colon tumors

Set up 1.5mL tubes, 1mL digestion mix (RPMI containing 200U/mL collagenase IV and 0.5U/mL DNase I) per mouse, fine scissors, PBS, EDTA (500mM), 70µm cell strainers, 50mL tubes, PBS containing 2mM EDTA, and FACS buffer (PBS + 3% FCS) (**Tables 1**–**3**). The colon tumors collected per mouse are transferred into a new 1.5mL tube filled with 300µL of digestion mix (RPMI containing 200U/mL collagenase IV and 0.5U/mL DNase I). Cut the tumor tissue into very small pieces in the digestion mix (briefly clean the scissors in PBS between samples to avoid contamination of the samples). Expect one minute of cutting as a reference value, but note that more tumors may require a longer cutting time. Be conscious that dissociating the tissue for too long can lead to congealment of the digested tissue and greatly reduce cell yield. Therefore, closely monitor the cell suspension and stop cutting when the pieces of tumor tissue are not getting finer but starting to coagulate. Subsequently, 700µL of the digestion mix (RPMI containing 200U/mL collagenase IV and 0.5U/mL DNase I) is added to each tube containing the finely cut tumor tissue and incubated on a thermal shaker for 45min at 37°C at 1200rpm. Next, add 20µL 500mM EDTA solution per 1mL digestion solution (final concentration of 10mM) and incubate for 5min at room temperature to separate cell clusters. Then pass the cell suspension through a 70µm cell strainer into a 50mL tube and wash the strainer once with 10mL PBS/2mM EDTA. Finally, centrifuge the cells for 5min at 400g (4°C), discard the supernatant, and resuspend the cell pellet in 1mL FACS buffer for counting (**Table 1**).

Besides in the tumor tissue, it is also possible to determine leukocyte cell populations in the colon. Since the enzymatic digestion described here is not applicable to colon tissue, the use of a different protocol is required. Recently, guidelines for the digestion and flow cytometry analysis of intestinal tissue have been published (40).

### Flow cytometric staining and analysis of tumor-infiltrating myeloid and lymphocyte cell populations

Set up 96-well V-bottom plates, Fc-block, FACS buffer (PBS + 3% FCS), antibodies, viability stain, 4% formaldehyde (FA) buffered and diluted in PBS to 2% (**Tables 1**–**3**). Prepare an antibody cocktail for analysis of tumor-associated leukocytes by diluting fluorescently conjugated antibodies specific for F4/80, CD90.2, CD45, Ly6C, CD11b, XCR1, PDCA1 (CD317), Ly6G, MHCII, CD103, CD64, SIRP1α, CD19, TCRβ, CD11c, and fixable viability stain (FSV) in FACS buffer (dilutions are listed in **Table 4**). Store in the dark at 4°C until use. Single cell suspensions of tumor cells are transferred at 2x10^6^ cells/well into a 96-well V-bottom plate, pelleted (centrifuge 5min at 300g, 4°C), and resuspended in 40µL Fc-block (diluted 1:20 in FACS buffer) for 15min at 4°C to prevent non-specific Fc-receptor mediated antibody binding. Thus, antibodies against CD16 and/or CD32 have to be incubated prior to the Fc-block. After Fc-blocking, the blocking solution is diluted by adding 100µl FACS buffer. Now centrifuge the cells for 5min at 300g and 4°C. Discard the supernatant and resuspend the cells in 40µl antibody cocktail and incubate for 20min in the dark at 4°C. Cells are washed two times with 200µl FACS buffer and resuspended in 100µl of 2% FA in PBS and incubated for 15min at room temperature for fixation. Fixation is stopped by adding 100µl PBS, centrifuge the cells (5min at 300g, 4°C) and add 200µl FACS buffer to wash the cells. Centrifuge again (5min at 300g, 4°C) and resuspend the cells in 100µl FACS buffer and keep them on ice or in the fridge at 4°C in the dark until measurement.

**Table 4:** List of dyes and antibodies used for flow cytometry.

Antibody/ Dye	Conjugate	Host/Isotype	Clone	Supplier	Dilution	Catalog Number
CD45pan	BV510	Rat IgG2b	30-F11	Biolegend	1:200	103138
Ly6C	BV570	Rat IgG2c, κ	HK1.4	Biolegend	1:500	128030
CD11b	BV605	Rat IgG2b, κ	M1/70	BD Biosciences	1:250	563015
XCR1	BV650	Mouse IgG2b, κ	ZET	Biolegend	1:500	148220
PDCA1/CD317	BV711	Rat IgG2b, κ	927	BD Biosciences	1:500	747604
Ly6G	BV750	Rat IgG2a, κ	1A8	BD Biosciences	1:250	747072
MHCII	BV786	Rat IgG2b, κ	M5/114.15.2	BD Biosciences	1:250	742894
CD103	Alexa 488	Hamster IgG	2E7	Biolegend	1:100	121408
CD64	PerCP-710	Mouse IgG1, κ	X54-5/7.1	ThermoFisher	1:500	46-0641-82
SIRP1α	PE-Cy7	Rat IgG1, κ	P84	Biolegend	1:500	144008
CD19	PE-Cy5	Rat IgG2a, κ	6D5	Biolegend	1:800	115510
CD11c	APC-R700	Hamster IgG2	N418	BD Biosciences	1:500	565872
F4/80	BUV737	Rat IgG2a, κ	T45-2342	BD Biosciences	1:500	749283
CD90.2	Pacific Blue	Rat IgG2a	53-2.1	Biolegend	1:1000	140306
FSV780	APC-Cy7			BD Biosciences	1:1000	565388
Fc Block (Anti-mouse CD16/CD32)		Rat IgG2b, κ	2.4G2	BD Biosciences	1:20	553142
True-Stain Monocyte Blocker				BD Biosciences	1:50	426103
Brilliant Stain Buffer Plus				BD Biosciences	1:12,5	566385

For data acquisition and manual analysis of spectral flow cytometry data, cells were acquired on the FACS Symphony™ of the Core Facility Flow Cytometry (CFFC) at the Research Center for Immunotherapy (Forschungszentrum für Immuntherapie, FZI) of the Medical Center of the Johannes Gutenberg-University Mainz. The configuration of the system can be found here: https://www.cffc.uni-mainz.de/symphony/. For acquisition, cells are stored in FACS buffer. Subsequent data analysis was performed using FlowJo v10.8.1 software.

## Results

### Tumor area measurement

**Figure 3:**
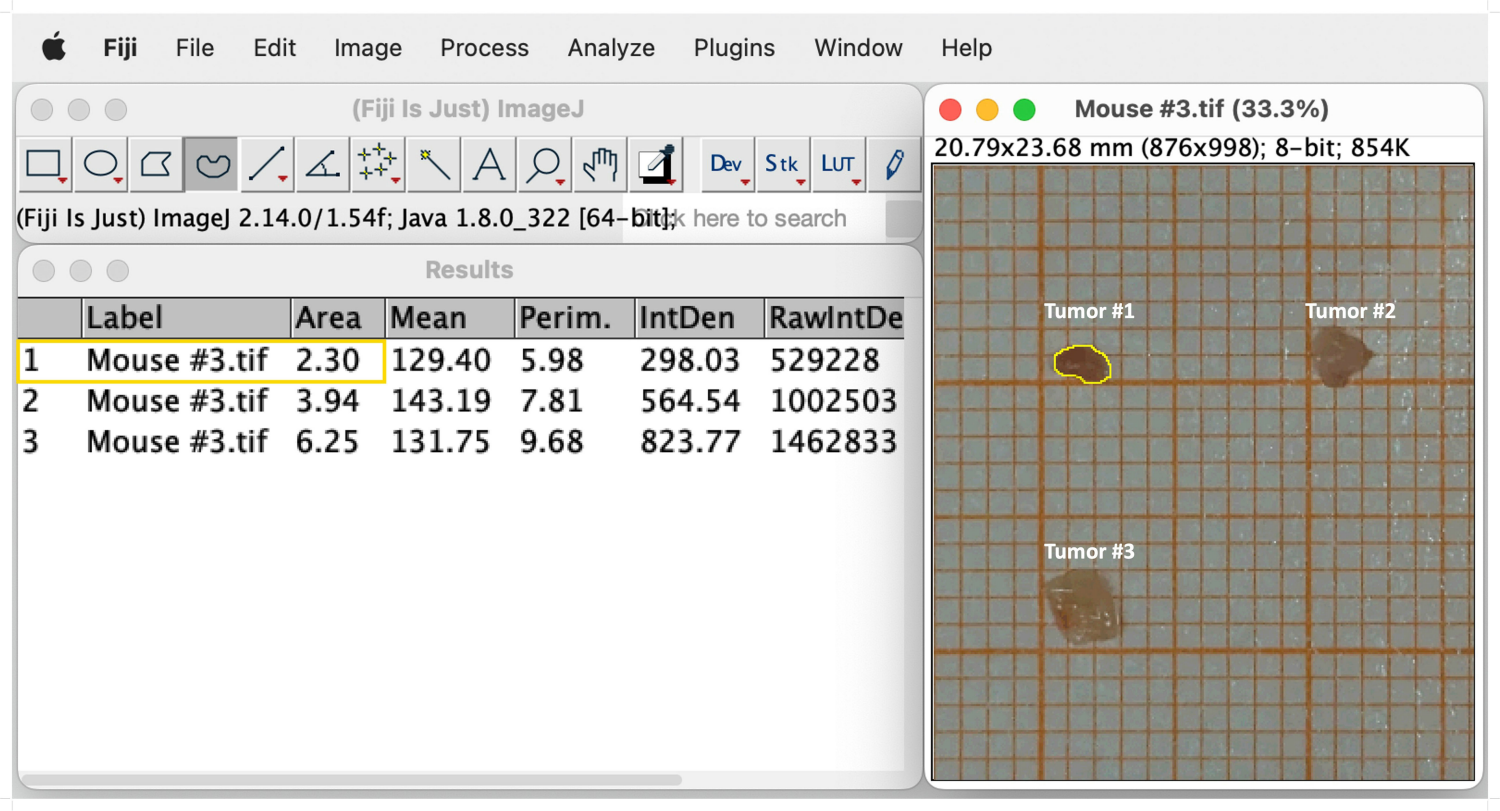
Representative example of digital tumor area measurement using Fiji software. Selection of the freehand option and drawing a line around the edges of the tumor (Tumor #1, yellow line). Tumor size is measured by choosing `Analyze´ and subsequently `Measure´. The measurements of the sizes appear in a separate window called `Results´. See text for details.

Tumors induced by the AOM/DSS protocol can display considerable differences in number between individual mice, which significantly affects absolute cell numbers in downstream analyses. Therefore, we recommend to normalize the total tumor cell count to the total tumor area, before calculating the absolute cell number of the individual leukocyte populations. This section provides a simple and straightforward approach to calculate the tumor area of AOM/DSS-induced tumors in mice. After sacrificing the mice on day 60 of the AOM/DSS protocol (**Figure 1**), isolated tumors are placed on laminated graph paper for subsequent quantification of tumor development. Digital tumor area measurement is performed using Fiji software (Version 2.14.0/1.54f) (41) (**Figure 3**). To ensure the most accurate area measurement possible, it is advisable to take the pictures with a high-resolution camera. After opening the image file in Fiji, change the image type to 8-bit Color by selecting ‘Image’, ‘Type’ and then ‘8-bit Color’. Next, calculate the average number of pixels per mm by choosing the ‘Straight’ selection and drawing along the length of one side of a mm square on the graph paper. Select ‘Analyze’ in the toolbar and ‘Measure’ from the pull-down menu. Repeat this step multiple times to ensure correct measurement of pixels. Then, select ‘Results’ and ‘Summarize’ to get the mean length of pixels measured for one mm. Subsequently, select ‘Analyze’ and ‘Set Scale’ to feed the program with the exact number of pixels per mm. Enter the previously calculated mean ‘Distance in pixels’ and set the ‘Known distance’ to one and the ‘Unit of length’ to mm. It is crucial to reset the scale for each image since the pixels per mm can vary from image to image and affect the area measurement. Finally, select the ‘Freehand’ option and precisely draw a line around the perimeter of each tumor (**Figure 3**). Then select ‘Analyze’ and ‘Measure’ to calculate the area of the tumors. Divide the total cell count for all tumors of each mouse by their respective combined tumor area and use the subsequent ratio (tumor cells/mm^2^) to calculate the absolute cell number of the individual leukocyte populations from their frequencies.

### Quantification of tumor burden and size

**Figure 4:**
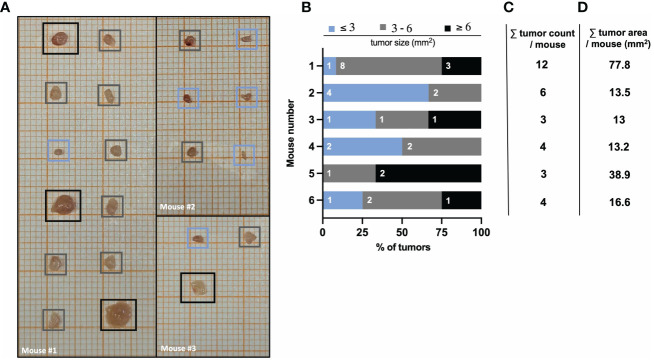
AOM/DSS protocol leads to heterogeneous tumor formation in wild type mice. **(A)** Representative images of isolated colon tumors from three different wild type mice at day 60 after AOM/DSS treatment. Color-coded boxes indicate the classification of tumor size as depicted in **(B)**. **(B)** Size distribution of AOM/DSS-induced tumors from six different wild type mice was determined *ex vivo* on day 60. Bar diagrams indicate the quantification of tumor development per mouse, based on tumor size in mm^2^ (area) and the number of tumors per size group. **(C)** Total tumor count and **(D)** total tumor area in mm^2^ per mouse corresponding to the mice listed in **(B)**.

The number and size of tumors can be quantified by macroscopic inspection and measurement using laminated graph paper. As described above, tumors isolated on day 60 of the AOM/DSS model are placed on laminated graph paper for subsequent tumor area determination (see ‘Tumor area measurement’ for precise instruction). The comparison of tumors from different (wild type) mice can reveal a strong heterogeneity in number and size (**Figure 4A**). Therefore, quantification of tumor formation is very important, especially when comparing mice with different genotypes, as the significance of tumor counting alone is limited. For example, mouse #3 and #5 both have a low total tumor count of 3 (**Figure 4C**), but when comparing the tumor area, mouse #5 has a tumor area three times larger than mouse #3 (**Figure 4D**). Hence, by classifying tumor sizes into multiple categories (e.g., 3mm, 3-6mm, 6mm), it is possible to obtain more meaningful information about tumor burden (**Figure 4B**). This represents a valuable readout of tumor development, since increasing tumor size in patients with colorectal cancer correlates positively with cancer stage, and the 5-year overall survival decreases significantly with increasing tumor size (42). Furthermore, in mice, tumor number and tumor size can be used to identify factors that regulate tumor initiation and progression. Variations in average tumor size can provide clues to factors involved in tumor progression. On the other hand, changes in the average number of tumors per animal should reflect factors that influence tumor initiation (43).

### High-dimensional flow cytometry of tumor-associated leukocytes

**Figure 5:**
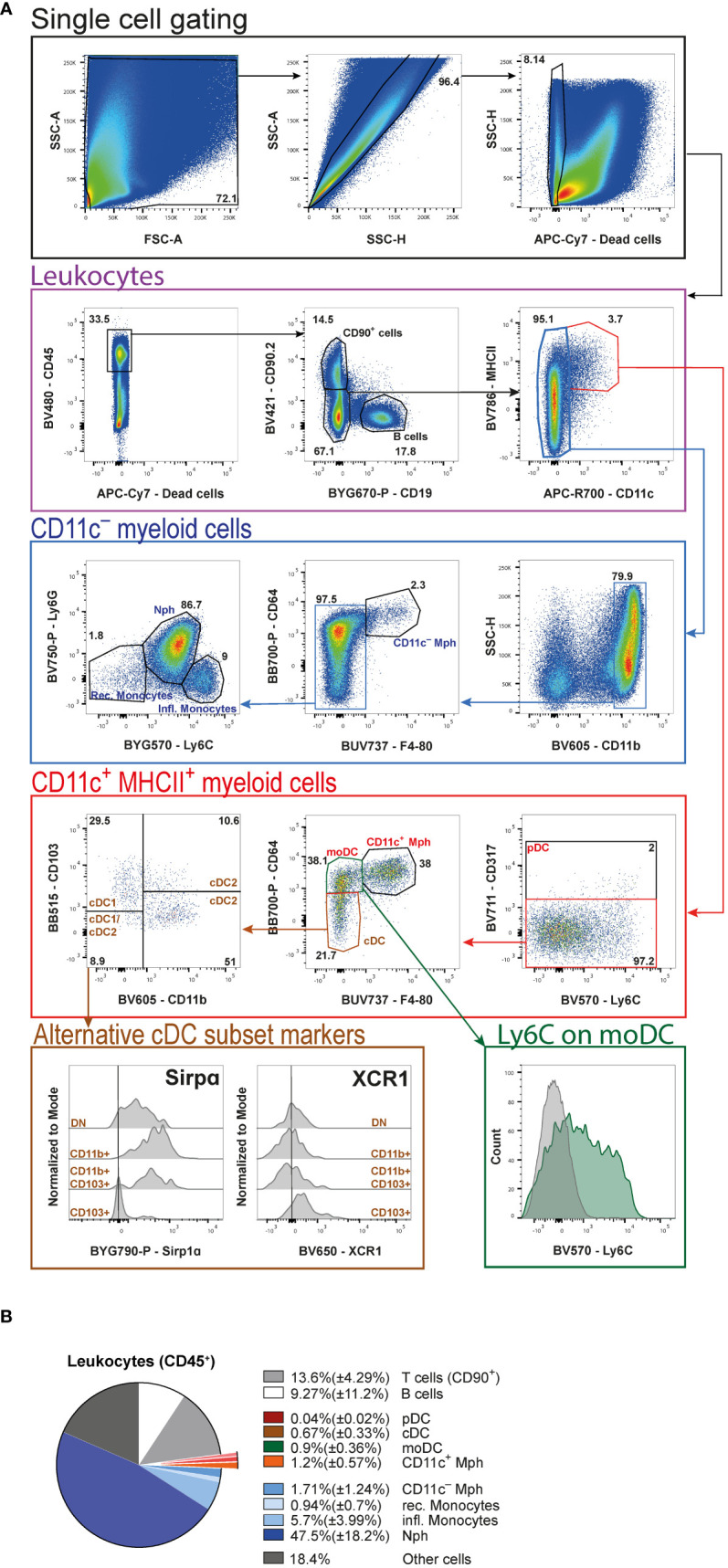
High-dimensional FACS analysis of tumor-associated leukocytes. **(A)** Gating strategy of merged tumor samples to identify the cellular composition of tumor-infiltrating leukocytes in AOM/DSS-induced CRC at day 60. Initial gating steps are organized into the identification of live single cells and the leukocytes therein. The subsequent analysis is divided into CD11c^+^ and CD11c^−^ myeloid cells. **(B)** Pie chart of the relative cellular composition among leukocytes, based on individual samples as gated in **(A)**. pDC (plasmacytoid DC), Mph (macrophages), moDC (monocyte-derived DC), cDC (conventional DC), Nph (neutrophils) rec. monocytes (recirculating monocytes), infl. monocytes (inflammatory monocytes).

For the flow cytometric analysis of tumor-infiltrating and -associated leukocytes (**Figure 5A**), we first gate on all cells to exclude any debris (**Figure 5A**, *black frame*). Next, doublets are excluded before gating on live cells and then CD45^+^ leukocytes (**Figure 5A**, *violet frame*). Separating live cells and subsequent leukocyte gating allows to calculate the total cell count of each population based on the live cells counted after digestion. To get a general idea of the number and distribution of lymphocytes, CD90.2 and CD19 are included as markers for T cells and B cells, respectively. Of note, some innate lymphoid cells (ILC) can also express the surface marker CD90.2. Double negative cells for CD90.2 and CD19 comprise all myeloid cells and are further subdivided into CD11c^+^ (which are all also MHCII^+^) and CD11c^-^ cells (**Figure 5A**, *violet frame*).

Proceeding with the CD11c^+^MHCII^+^ double-positive myeloid cells (**Figure 5A**, *red frame*), first pDC are defined by the expression of CD317. The CD317^−^ cells are then subdivided into CD11c^+^ Mph, which are CD64^+^F4/80^+^, CD64^+^F4/80^−^ monocyte-derived DC (moDC) and CD64^−^F4/80^−^ cDC. Among DC, cDC subsets are identified based on their expression of CD103 and CD11b (cDC1: CD103^+^CD11b^−^, cDC2: CD103^+^CD11b^+^ and CD103^−^CD11b^+^, and cDC1/cDC2: CD103^−^CD11b^−^). The alternative markers, SIRPα and XCR1, commonly used to distinguish cDC1 and cDC2, prove less reliable to identify the different cDC subsets in the tumors, as seen in the histograms (**Figure 5A**, *brown frame*). We also include Ly6C in the antibody cocktail to demonstrate that moDC display a heterogeneous expression of this marker (**Figure 5A**, *green frame*).

From the CD11c^−^ leukocytes (**Figure 5A**, *blue frame*), cells are first positively selected for the expression of CD11b. Among these cells we are able to identify CD11c^−^ Mph according to their CD64 and F4/80 expression. Finally, the remaining F4/80^−^ cells are subdivided into Ly6G^+^L6C^int^ neutrophils (Nph), Ly6C^−^Ly6G^−^ recirculating, and Ly6C^+^Ly6G^−^ inflammatory monocytes.

Among all living cells in colitis-associated tumors 41.9%( ± 8.59%) are leukocytes. Statistical analysis of these leukocytes (**Figure 5B**) reveals that neutrophils (Nph) represent the biggest cell population [47.5%( ± 18.2%)], followed by CD90^+^ T cells [13.6% ± 4.29%)] and B cells [9.27%( ± 11.2%)]. As far as Mph are concerned, the CD11c^+^ [1.2%( ± 0.57%)] and the CD11c^−^ [1.71%( ± 1.24%)] populations are comparable in relative size. Within the DC compartment, moDC [0.9%( ± 0.36%)] represent the largest population, followed by cDC [0.67%( ± 0.33%)]. pDC are barely detectable at a total population size of 0.04% ( ± 0.02%). Amongst monocytes, the frequency of inflammatory monocytes [5.7%( ± 3.99%)] is more than four times that of recirculating monocytes [0.94%( ± 0.7%)] (**Figure 5B**).

## Discussion

Here, we provide a fast and simple step-by-step protocol to isolate colorectal tumors induced by the AOM/DSS model in mice and to characterize the leukocyte, particularly myeloid, tumor cell infiltrate using high-dimensional flow cytometry. Initial isolation of the tumors is easy to perform by cutting carefully around the cancerous tissue. The subsequent gentle enzymatic digestion of the tumor tissue we describe to produce a single cell suspension ensures a high yield of living CD45^+^ leukocytes [41.9%( ± 8.59%)]. The procedure is simple and easy to follow without requiring the use of pricey kits and equipment, which often affect epitopes essential for leukocyte subset identification, or laborious density gradient centrifugation. Notably, we have not yet been able to detect any cleaved epitopes on myeloid cells using this digestion method. It therefore allows a comprehensive and reproducible flow cytometric analysis of the distribution of the different myeloid cell populations, including DC, Mph and Nph, in tumors of the murine AOM/DSS model. Beyond the high-dimensional flow cytometry analysis presented here, the total leukocytes or specific myeloid subpopulations extracted and purified with this protocol can be used for unbiased single-cell RNA sequencing and proteomics. Tumor-infiltrating leukocytes are a very heterogeneous population and consequently their detailed phenotypic analysis by flow cytometry requires an extensive panel of cell type-specific antibodies and fluorochromes. We established a 16-surface marker antibody panel to first separate B and T lymphocytes from myeloid cells and then further distinguish between the different DC (cDC, pDC, moDC) and Mph (CD11c^+^ and CD11c^-^) subsets, Nph and monocytes. It is worth noting that our chosen T cell marker CD90.2 can also be expressed by a subpopulation of ILC and is not an exclusive T lymphocyte marker. However, this protocol is designed to allow easy adjustment of the flow cytometry antibodies to customize the staining panel.

DC, including cDC and pDC, are sentinels of the immune system and cDC represent its professional APC. Our analysis of the cancerous tissue shows that cDC constitute around 0.67%( ± 0.33%) of all leukocytes. They play a decisive role in priming tumor-specific T cells in the draining lymph nodes and thus contribute to induction of anti-tumor immunity. Intestinal cDC can be divided into two main populations based on their XCR1 and SIRPα (CD172a) expression, respectively. XCR1-expressing cDC1 are CD103^+^CD11b^-^ and are known for their role in combating intracellular pathogens and tumors. They polarize CD8^+^ T cells and are specialized in cross-presentation, a process in which exogenous antigens are processed and presented on MHCI molecules, and cDC1 are therefore important for self-tolerance in steady state. Furthermore, they play an essential role in the induction of tumor specific CD8^+^ cytotoxic T cells (2). cDC2 are more heterogeneous, with two main cDC2 populations existing in the intestine. Both can be identified by their expression of SIRPα and CD11b, but they differ regarding their expression of CD103. In terms of gene expression, intestinal CD11b^+^CD103^+^ cells belong to cDC2 and are known to be involved in CD4^+^ Th17 or Treg cell differentiation (2). CD11b^+^CD103^-^ cDC2 play an important role in the induction of CD4^+^ Th1, Th2, and Th17 cells and are less capable to induce Treg cells (3). Although intestinal cDC subsets are commonly classified using XCR1 and SIRPα, our analysis revealed that tumor-infiltrating cDC are rather heterogeneous with respect to the expression of these markers. We therefore propose that tumor-infiltrating cDC1 and cDC2 are more clearly described using CD11b and CD103. By their unique expression of CD317 (PDCA-1) we identified a minor population [0.04%( ± 0.02%)] of all leukocytes) of pDC in the tumor tissue. Despite its small size, the role of pDC in cancer cell killing can be crucial because they have the ability to cross-prime naïve CD8^+^ T cells by transferring antigen to cDC (4, 5). However, it was also shown that pDC can act tolerogenic by inhibiting tumor-directed immune responses, thus leading to tumor progression (44).

Moreover, our staining panel enables the identification of moDC among the CD11c^+^MHCII^+^ cells. They are CD64^+^F4/80^–^ and represent around 0.9% ( ± 0.36%) of all infiltrating tumor leukocytes. Here, our analysis reveals that moDC are heterogeneous for Ly6C expression and are more clearly identified using CD64 and F4/80. MoDC are mainly generated in peripheral tissues under inflammatory conditions and are resident in non-lymphoid tissues like the skin, the lung, and the intestine. They are implicated in the generation of Treg cells, thus acting immunosuppressive in cancer (4). Indeed, it was already shown that a low moDC count in the blood of CRC patients correlates positively with reduced metastasis (45).

Another notable CD11c^+^MHCII^+^ myeloid cell population are F4/80^+^CD64^+^ Mph (around 1.2%( ± 0.57%) of all infiltrating leukocytes). Intestinal Mph are essential in establishing and maintaining gut homeostasis as they produce a variety of cytokines and other mediators to maintain proliferation of epithelial cells (15). Traditionally, Mph are classified in pro-inflammatory (M1-like) or anti-inflammatory (M2-like) cells (23, 46). Nevertheless, in colon cancer Mph cannot be easily classified as M1 or M2 and rather display a remarkable dichotomy. Recently, C1q and SPP1 emerged as suitable surface markers to distinguish Mph subsets in colorectal cancer (13). Tumor angiogenesis, cell migration, extracellular-matrix receptor interaction, and tumor vasculature pathways are enriched in SPP1^+^ Mph, whereas complement activation and antigen processing and presentation pathways are enriched in C1q^+^ Mph (13). Furthermore, only C1q^+^ Mph could be identified in the colon mucosa of ulcerative colitis patients and healthy individuals (47), whereas SPP1^+^ Mph were largely absent in non-cancer tissues, suggesting a unique function in the CRC tumor environment (13). Of note, patients with C1q^high^ and SPP1^low^ Mph gene signatures had the best prognosis, whereas the opposite was seen in patients with C1q^low^ and SPP1^high^ Mph gene signatures (13). Therefore, it would be interesting to include C1q and SPP1 in future experiments allowing a more detailed analysis of the different Mph subsets, since they seem to play an essential role especially in CRC and are linked to the overall survivability.

In addition, we also detect a CD64^+^F4/80^+^ Mph population among the CD11c^–^ cells. These Mph highly express CX3CR1, are enriched in the muscularis, and are thought to be key players in regulating gastrointestinal motility through direct communication with enteric neurons (48). While muscularis Mph are indispensable for intestinal homeostasis and disease and can secrete IL-1, IL-4, and TNF, which leads to enteric glia cell activation (49), their role in CRC remains elusive.

The most prominent immune cell population in our data set are CD11b^+^Ly6C^int^Ly6G^+^ Nph (47.5%( ± 18.2%)). It is already known that Nph play a dual role in CRC (50). Originally, it was thought that Nph mediate an anti-tumorigenic effect, but then it has been revealed that so-called tumor-associated Nph have a tumor-supportive function (50). The plasticity between tumor- suppressive (N1 phenotype) and -supportive (N2 phenotype) Nph is regulated by TGF-β and INF-γ signaling. Moreover, the Nph-to-lymphocyte ratio is a well-defined predictive marker for CRC patients, as a high ratio is associated with poor outcome following hepatic resection for liver metastases (51). From a technical perspective, Nph are a sensitive cell population with high turnover, making it difficult to study these cells *ex vivo*. Therefore, rapid cell extraction is imperative, one of the main advantages of the protocol described here. Another CD11c^–^ myeloid cell population identified by their expression of CD11b and Ly6C that plays an important role in CRC are monocytes. According to their expression of Ly6C, it is possible to distinguish between recirculating (Ly6C^neg^) and inflammatory (Ly6C^+^) monocytes. Like the Nph-to-lymphocyte ratio also the monocyte-to-lymphocyte ratio is a prognostic factor in CRC patients. Monocytes are recruited as inflammatory cells to directly kill malignant cells and are able to induce cancer cell apoptosis (52).

In conclusion, we present a user-friendly protocol that enables rapid extraction and subsequent high-dimensional flow cytometric analysis of tumor-associated leukocytes from AOM/DSS-induced colorectal cancer in mice. Although this protocol focuses on flow cytometric analysis, purified cells can also be used for further analyses, such as unbiased single-cell RNA sequencing or mass spectrometry.

## Data availability statement

The original contributions presented in the study are included in the article/supplementary materials, further inquiries can be directed to the corresponding author/s.

## Ethics statement

The animal study was approved by Landesuntersuchungsamt Rheinland-Pfalz. The study was conducted in accordance with the local legislation and institutional requirements.

## Author contributions

CE: Conceptualization, Formal analysis, Investigation, Methodology, Writing – original draft, Writing – review & editing. JFV: Conceptualization, Formal analysis, Investigation, Methodology, Writing – original draft, Writing – review & editing. VL: Conceptualization, Formal analysis, Investigation, Methodology, Writing – original draft, Writing – review & editing. BEC: Conceptualization, Funding acquisition, Investigation, Resources, Supervision, Writing – original draft, Writing – review & editing. NH: Conceptualization, Funding acquisition, Investigation, Resources, Supervision, Writing – original draft, Writing – review & editing.
